# Physiological stress and Hendra virus in flying-foxes (*Pteropus* spp.), Australia

**DOI:** 10.1371/journal.pone.0182171

**Published:** 2017-08-02

**Authors:** Lee McMichael, Daniel Edson, Craig Smith, David Mayer, Ina Smith, Steven Kopp, Joanne Meers, Hume Field

**Affiliations:** 1 University of Queensland, School of Veterinary Science, Gatton, Queensland, Australia; 2 Biosecurity Queensland, Department of Agriculture and Fisheries, Brisbane, Queensland, Australia; 3 Department of Agriculture and Water Resources, Canberra, Australian Capital Territory, Australia; 4 Department of Agriculture and Fisheries, Brisbane, Queensland, Australia; 5 Australian Animal Health Laboratory, Geelong, Victoria, Australia; 6 EcoHealth Alliance, New York, New York, United States of America; Deutsches Primatenzentrum GmbH - Leibniz-Institut fur Primatenforschung, GERMANY

## Abstract

Pteropid bats (flying-foxes) are the natural reservoir of Hendra virus, an emergent paramyxovirus responsible for fatal infection in horses and humans in Australia. *Pteropus alecto* (the Black flying-fox) and the paraphyletic *P*. *conspicillatus* (the Spectacled flying-fox) appear to be the primary reservoir hosts. Previous studies have suggested that physiological and ecological factors may underpin infection dynamics in flying-foxes, and subsequent spillover to horses and in turn humans. We sought to examine temporal trends in urinary cortisol concentration in wild Australian flying-fox populations, to elucidate the putative relationship between Hendra virus infection and physiological stress. Pooled and individual urine samples were non-invasively collected from under roosting flying-foxes at two latitudinally disparate regions in the eastern Australian state of Queensland. Hendra virus detection, and (in individual urine samples) sex and species determination were PCR-based. Urinary cortisol measurement used a validated enzyme immunoassay. We found no direct correlation between increased urinary cortisol and Hendra virus excretion, but our findings do suggest a biologically plausible association between low winter temperatures and elevated cortisol levels in *P*. *alecto* in the lower latitude Southeast Queensland roosts. We hypothesize an indirect association between low winter temperatures and increased Hendra virus infection and excretion, mediated by the physiological cost of thermoregulation. Our findings and our approach are directly relevant to elaboration of the disease ecology of Nipah virus and other emerging henipaviruses in bats. More broadly, they inform investigation of emerging disease infection dynamics across the wildlife/livestock/human interface.

## Introduction

Bats of the genus *Pteropus* (Chiroptera), commonly known as flying-foxes, have been identified as the natural host of Hendra virus, a potentially fatal zoonotic paramyxovirus that causes mortality or morbidity in horses and humans in Australia [1, 2]. Of the four *Pteropus* species endemic to mainland Australia, there is accumulating evidence that the Black flying-fox (*Pteropus alecto)* and the paraphyletic Spectacled flying-fox (*P*. *conspicillatus*) [3] are primary reservoir hosts of Hendra virus [4–7].

Previous studies of *Pteropus* species have suggested that physiological and ecological factors may constitute risk factors for Hendra virus infection [8–11]. ‘Physiological stress’ is often poorly defined and loosely interpreted, presenting challenges for its robust measurement and comparative assessment. However, there are several well documented approaches to its assessment in the mammalian system, including assessment of endocrine and immune system function, oxidative stress and cellular senescence [12–16].

Assessment of glucocorticoid hormones is a recognised and robust methodology for investigating the putative association between physiological stress, infection and disease in wildlife species [17]. Glucocorticoid hormones are fundamental regulators of energy balance in mammalian species, and elevations in these hormones precipitate a stress response which modulates fertility, metabolic balance and immune function [12]. However, while the release of adrenal glucocorticoids can be crucial to survival when an acute stressor is present, chronically elevated or exceptionally high levels of glucocorticoids are usually detrimental to health [18].

Plasma glucocorticoid measurement has been employed in a number of chiropteran studies [18–21], and cortisol is recognised to be the primary plasma glucocorticoid in *Pteropus* species [22, 23]. More recently, McMichael *et al*., [24] demonstrated the under-roost collection of urine and the immunoassay of pooled urine samples for cortisol as a robust tool for the assessment of stress in wild Australian flying-foxes, correlating elevated plasma and urinary cortisol concentrations in response to physiological stress, and establishing urinary cortisol profiles for the four species occurring on mainland Australia. In a subsequent study, Edson *et al*., [25] reported that anthropogenic disturbance of flying-fox roosts did not result in urinary cortisol fluctuations that were significantly different from background levels, nor did it precipitate any detectible increase in Hendra virus excretion prevalence. The study did however find a small positive association between Hendra virus excretion status and urinary cortisol concentration.

Understanding Hendra virus infection dynamics in flying-foxes and the contributing risk factors is fundamental to elaborating the disease ecology of the virus and thus effective exposure risk management in horses and humans. In this study, we sought to examine temporal trends in urinary cortisol concentration in two disparate wild Australian flying-fox populations, and elucidate the putative relationship between Hendra virus infection and physiological stress.

## Materials and methods

### Study sites, animals and ethics

Roost sites were identified using information from the Queensland Department of Environment and Heritage Protection (EHP) and the (then) Queensland Centre for Emerging Infectious Diseases (QCEID). Fieldwork was conducted under the Queensland Department of Agriculture, Fisheries and Forestry Animal Ethics Committee Permit SA 2011/12/375, and Department of Environment, Heritage and Protection Scientific Purposes Permits WISP05810609 and WISP14100614.

**Fig 1:**
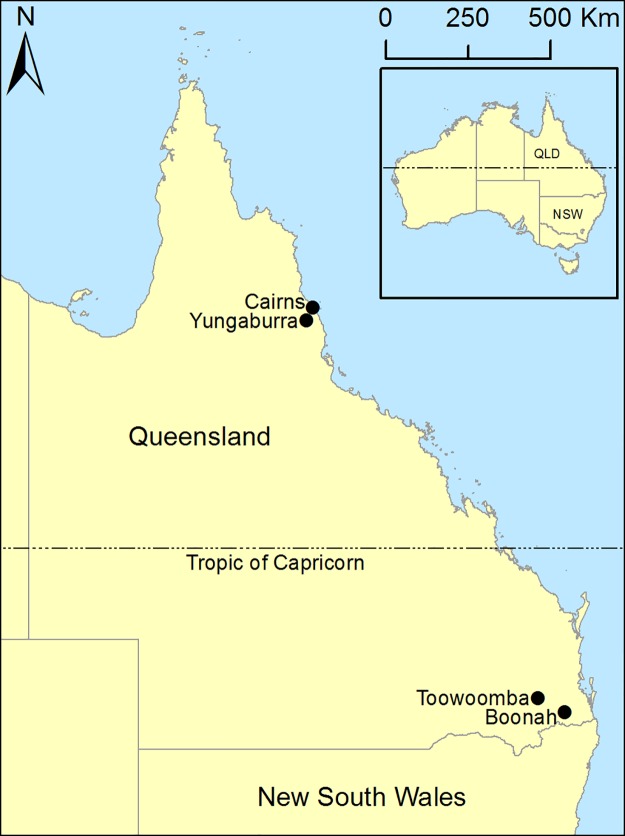
Map of Queensland (Australia) illustrating the disparate SEQ and FNQ regions and the locality of sampled flying-fox roosts.

Urine samples were collected from beneath roosting flying-foxes at two roosts in each of two disparate regions in the eastern Australian state of Queensland between January 2012 and May 2014: in Southeast Queensland (SEQ) (latitude -27°), at roosts in the rural locations of Boonah and Toowoomba; and in Far North Queensland (FNQ) (latitude -17°) at roosts in the rural location of Yungaburra and the urban location of Cairns (Fig 1). As flying-foxes are highly mobile animals, samples from either roost within the SEQ and FNQ regions can reasonably be considered representative of a single regional population. Total population and species composition counts were estimated by conducting counts of roosting individual animals throughout the roost site, specifically, by counting the number of animals per tree multiplied by number of trees throughout the roost. Total population was additionally estimated during nightly fly-out, based on fly-out density and time of complete evacuation of the roost site. Reproductive behaviour throughout the roost was observed and recorded during each sampling event, including male/female interactions and vocalisations, and the number of females with visually obvious pregnancies and dependent pups.

### Sample and data collection

Both pooled and individual urine samples were collected from the SEQ Boonah roost; pooled samples only were collected from the FNQ Cairns and Yungaburra roosts; and individual samples only were collected from the SEQ Toowoomba roost. Pooled urine collections were undertaken as a shared component of related studies reported elsewhere [7, 25]. A total of 2208 pooled samples were collected; 1112 from the Boonah roost during 30 sampling events from January 2012 to May 2014; 826 from the Cairns roost during 21 sampling events from May 2012 to June 2014; and 270 from the Yungaburra roost during eight sampling events from February 2012 to April 2013. A total of 464 individual samples were collected at the Boonah and Toowoomba roosts during nine sampling events from April 2013 to May 2014.

Collection of pooled urine samples was performed as per McMichael *et al*., [24] using plastic sheeting placed under trees in which flying-foxes were roosting. The target sample size and volume was 30–60 x 1mL samples collected from ten sheets spaced opportunistically throughout accessible roost. Sheets were placed either pre-dawn on the morning of collection prior to animals returning to roost, or the previous evening after animals had left the roost. In the hour post-dawn, three or four pooled samples (each typically comprising 10–20 urine droplets) were methodically collected from discrete sections of each sheet to minimise an individual bat's potential contribution to multiple pools. Samples were immediately placed on ice in the field for transport to the Biosecurity Sciences Laboratory (BSL) in Brisbane, where they were stored at -80°C until testing. The pooled samples from all ten sheets contained urine from an estimated 50–200 individuals depending on flying-fox roosting density. Pooled samples were used to provide summary roost population estimates of urinary cortisol concentration over space and time; no interpolation of the characteristics of individuals contributing to the pool was made.

Collection of individual animal under-roost urine samples was typically performed using transects of plastic sheeting (measuring 1 m x 40 m) placed parallel to one another approximately 5 m apart, through accessible areas of the roost. The number of transects varied with roost size and accessibility, with 4 to 5 x 40 m transects at Boonah, and 10 x 5 m transects at the smaller Toowoomba roost. Individual animal urine target sample volume was 50–200 μl. The number of samples collected at a single sampling event ranged from 46 to 109. Individual animal urination events were identified by spatial separation of at least one meter between urine splatter patterns. This criterion was based on simulated urine splatter patterns, wherein 10 ml volumes of coloured water were expressed from a syringe from a 5 m height onto plastic sheeting, producing an average splatter size of 0.5 m x 0.5 m. The urine sample was taken from one droplet or 2–3 closely associated droplets from an individual urination event. Samples were not collected when roosting density was so high as to preclude confident identification of an individual urination event. This occurred on a limited number of occasions associated with large influxes of Little red flying-foxes, and only in sections of the roost. Samples were collected in the hour post-dawn, and transported and stored as for pooled samples.

The climatic variables of minimum and maximum daily temperature and daily rainfall for the SEQ and FNQ roosts from January 2012 to June 2014 were collated from the Australian Bureau of Meteorology [26]. To avoid confounding, the months of February and March associated with peak mating activity and natural elevations in urinary cortisol [27–29] were excluded from this analysis.

### Hendra virus detection

PCR-based Hendra virus detection was undertaken on the individual animal under-roost urine samples. Immediately post-collection, 50 μl of each urine sample was added to a corresponding 130 μL of Magmax lysis buffer (AM 8500) to preserve RNA for extraction and Hendra virus PCR analysis. The remainder of the sample was maintained neat for hormone analysis. Samples for PCR analysis underwent total nucleic acid extraction in the BSL Physical Containment Level 3 (PC3) laboratory using the Kingfisher automated extraction system and the MagMax viral RNA Isolation Kit (Catalogue number AMB18365) according to manufacturer’s instructions. RNA extracts (5 μL) were added to 20 μL reaction mix and assayed in duplicate for Hendra virus genome using a quantitative TaqMan RT-PCR targeting the Hendra virus M gene [30], using the Applied Biosystems 7500 Fast Real Time PCR System. Results were analysed by AB7500 software version 2.0.6. A mean cycle threshold value of <40 was defined as a positive result. Hendra virus detection on the pooled under-roost samples was undertaken as a component of related studies [7, 25] using the same technique.

### Species and sex determination

A novel PCR approach using the total nucleic acid extracted for the Hendra virus assay was used to establish the species and sex of the individual animal samples. The cytochrome B gene was targeted for species differentiation, and the SRY gene of the Y chromosome for sex differentiation. Extracted nucleic acid underwent a quantitative TaqMan RT-PCR using the TaqMan Universal Master Mix II, no UNG (product number 4440040). Primers (S1 Text) were supplied by Sigma Aldrich, Australia, and used at 10 pmol/μL; probes were supplied by Applied Biosystems Custom Oligo Synthesis Service, USA, and used at 10 pmol/μL. The thermal cycling was performed as follows: 50°C for 2 minutes, 95°C for 10 minutes, followed by 50 cycles of 95°C for 15 seconds, and 60°C for 60 seconds. Assay validation was performed on urine samples of known sex and species from captured individual animals and demonstrated 100% accuracy of detection for all species and both sexes (data not shown). Species and sex determination was not undertaken on pooled urine samples.

### Cortisol concentration

Urinary cortisol concentration was measured using Caymen Chemical Company Cortisol Enzyme Immunoassay (EIA), (Product number 500360), employing a synthetic cortisol-specific mouse monoclonal antibody (4-pregnen-11β, 17, 21-TRIOL-3, 20-DIONE). The assay was validated by the manufacturers for human urine and plasma, and mouse faeces, with minimum detectable cortisol concentration of 0.2 ng/ml. McMichael *et al*., [24] reported an adapted methodology in Australian flying-foxes and showed it to provide a robust assessment of stress in these species. We use that methodology in this study to measure urinary cortisol concentration profiles in *P*, *alecto* and *P*. *conspicillatus*, the two species in which Hendra virus RNA was detected. Briefly, duplicate pooled urine samples were diluted 1:10 in EIA kit buffer and assayed for cortisol according to manufacturer instructions. Absorbances were read at 405 nm with a Thermo Scientific Multiscan Ascent plate reader using Ascent software (Version 2.6). When measurements fell outside the range of 20–80% B/B0, samples were diluted and re-assayed to ensure the measurements fell within the linear range of the standard curve. Cortisol concentration was determined by analysis of absorbance values using MyAssays Analysis Software Solutions Cortisol "Four Parameter Logistic Curve" online data analysis tool (MyAssays Ltd., 2012, http://www.myassays.com/four-parameter-logistic-curve.assay). McMichael *et al*., [24] examined the effect of storage and treatment conditions on cortisol concentration in their initial validation of the assay, and found neither temperature nor buffer treatment had any significant effect on detected cortisol concentration.

### Statistical analyses

Generalised linear mixed models (GLMM) [31] were used primarily to test the significance of the various interactions and to obtain valid standard errors for the points. The observed patterns amongst GLMM means were further investigated using regression analyses, and these form the basis for the figures and interpretations. Analyses were undertaken using Genstat [32].

The pooled urinary cortisol, the individual urinary cortisol and Hendra virus data were subjected to separate unbalanced generalised linear mixed model (GLMM) analyses. The normal or log normal distribution was adopted as appropriate for continuous variables, and the binomial distribution and logit link for binary variables. Cortisol data proved to be positively skewed with heterogeneous variance and were transformed using the natural log (ln) prior to analysis. The residual plots proved to be approximately normal.

In the pooled urinary cortisol data analysis, the response variable was urinary cortisol concentration. The fixed effects were year, month, region, total population, species composition, minimum temperature on the day of sampling, and maximum temperature and daily rainfall on the day prior to sampling. Interaction terms were limited to three-way interactions for model parsimony. Sampling day was the additional random effect. Additional linear and nonlinear regression analyses of the effects of the environmental variables of minimum and maximum temperature and rainfall on urinary cortisol measurements were performed.

Two analyses were performed on the individual urinary data. Firstly, the response variable was urinary cortisol concentration, and secondly, the response variable was Hendra virus detection. For each analysis, the fixed effects were month, sex, reproductive activity and their (three-way) interactions. Sampling day was the additional random effect.

Data were plotted and trends estimated by sampling day, and then were grouped into monthly data for temporal regression analysis. For the cortisol results, the direct back-transformation from the natural log was adopted for presentation, giving geometric means. Annual patterns for the variables were initially investigated with symmetrical Fourier curves, however Gaussian or double-Gaussian curves were subsequently adopted, as these allow a more general shape plus a direct test of the significance of any second annual peak.

## Results

### Roost site observations

**Fig 2:**
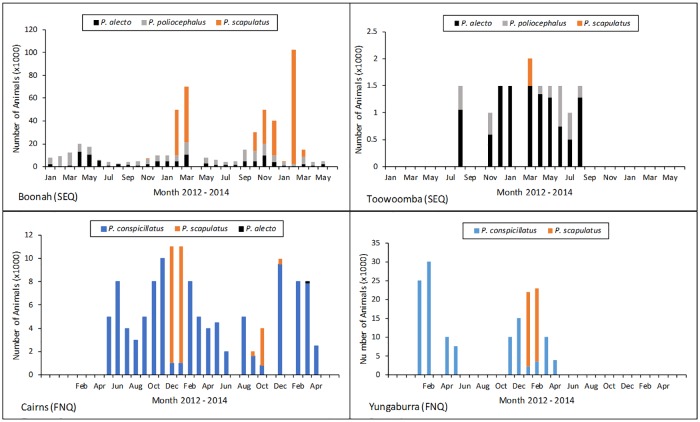
Total population and species composition at sampled roosts between 2012 and 2014.

Sampled roosts variously contained all four flying-fox species occurring on mainland Australia, namely *P*. *alecto* (the Black flying-fox), *P*. *poliocephalus* (the Grey-headed flying-fox), *P*. *conspicillatus* (the Spectacled flying-fox), and *P*. *scapulatus* (the Little red flying-fox). In SEQ, the Boonah roost consisted primarily of *P*. *alecto* and *P*. *poliocephalus* in varying ratios, but total population of these species never exceeded an estimated 20,000 individuals. Large numbers of *P*. *scapulatus* (up to 100,000 individuals) were periodically present at the roost site. The Toowoomba roost was consistently smaller than the Boonah roost and consisted primarily of *P*. *alecto* and *P*. *poliocephalus* in varying ratios with total population ranging from 1,000 to 2,000 with an influx of approximately 500 *P*. *scapulatus* in March 2013 (Fig 2).

In FNQ, the Yungaburra roost consisted primarily of *P*. *conspicillatus* with total population ranging from 4,000 to 30,000, with an influx of approximately 20,000 *P*. *scapulatus* in February and March 2013. The Cairns roost was consistently smaller than Yungaburra, and consisted primarily of *P*. *conspicillatus* with total population ranging from 800 to 11,000, with an influx of approximately 10,000 *P*. *scapulatus* in December 2012 and January 2013, approximately 3,000 *P*. *scapulatus* in October 2013, and approximately 100 *P*. *alecto* in March 2014 (data from: Field *et al*., [7]) (Fig 2).

At the SEQ roosts, peak mating activity of *P*. *alecto* and *P*. *poliocephalus* was observed in March each year. At the Boonah roost, 95% adult females were palpably pregnant from June onwards in both 2013 and 2014 [33], and visibly pregnant from August to October. First pups were observed in September, dependent pups observed from September to April, and independent juveniles first observed in April. At the FNQ roosts, mating of *P*. *conspicillatus* was observed in February and March each year. Females were palpably pregnant in June (McMichael *et al*., unpublished data), and first pups were observed in September.

Temperatures in SEQ ranged from -4°C to 44.6°C over the study period, with the highest mean monthly temperatures recorded annually in December and January, and the lowest mean monthly temperatures recorded in July and August. Temperatures in FNQ ranged from 9.8°C to 34.9°C over the study period, with the highest mean monthly temperatures recorded annually between December and February, and the lowest mean monthly temperatures in July and August. Daily rainfall in SEQ ranged from 0 mm to 255 mm over the study period, with the highest mean monthly rainfalls recorded annually in December and January, and the lowest mean monthly rainfalls between July and September. Daily rainfall in FNQ ranged from 0 mm to 241 mm over the study period, with the highest mean monthly rainfalls recorded annually between January and March and the lowest mean monthly rainfalls in August.

### Pooled urine samples

**Fig 3:**
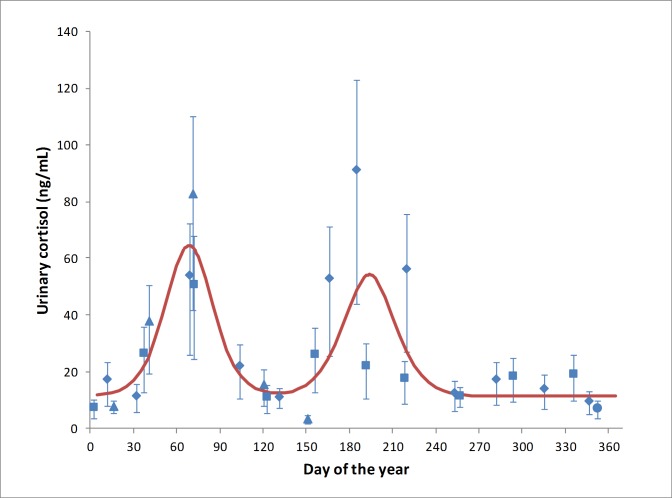
Mean urinary cortisol measurements derived from 1112 pooled urine samples collected at 30 sampling events across the 29-month study period at Boonah, SEQ, 2011 - ●; 2012 - ♦; 2013 - ■; 2014 - ▲, demonstrating an estimated overall annual population trend of urinary cortisol, (R^2^ = 0.59).

**Fig 4:**
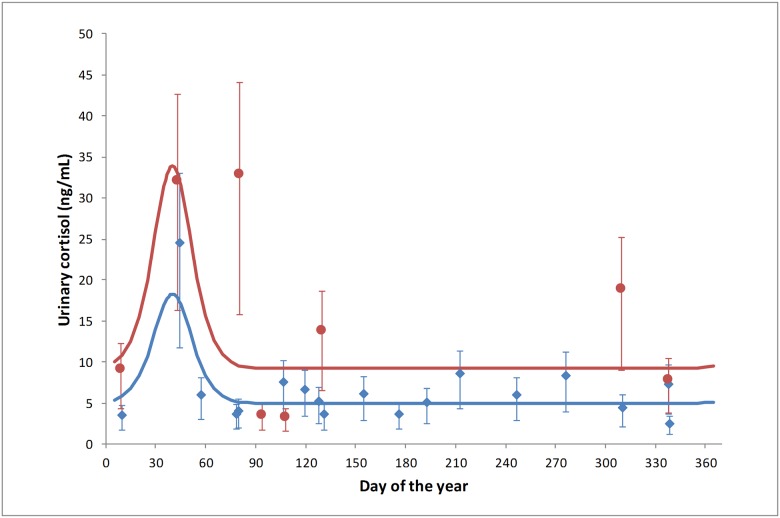
Mean urinary cortisol measurements derived from 826 (Cairns) and 270 (Yungaburra) pooled urine samples collected at 21 and 8 sampling events, respectively, across the 29-month study period in FNQ, demonstrating an estimated overall annual population trend of urinary cortisol for Cairns - ♦; Yungaburra - ■ (R^2^ = 0.46).

Pooled animal samples were collected from the SEQ Boonah roost and both FNQ roosts (Cairns and Yungaburra) (S1 Table). Significantly different urinary cortisol patterns were evident by GLMM analysis between the SEQ and FNQ regions (p < 0.001), and between months within both regions (p < 0.001). In SEQ, the overall annual pattern of urinary cortisol concentration showed two significant annual peaks, the first at day 68.7 ± 22.7 (late February/March, being late Summer/early Autumn in the southern hemisphere), and the second peak at day 193.8 ± 24.0 (July, being Winter in the southern hemisphere) (Fig 3). In FNQ, the overall annual pattern of urinary cortisol concentration showed only one significant annual peak, at day 40.1 ± 14.7 days (February, being late summer in the southern hemisphere) (Fig 4).

**Fig 5:**
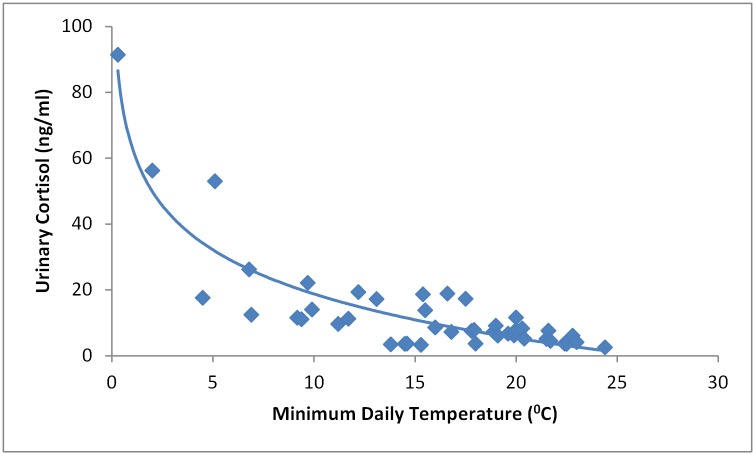
Pooled urinary cortisol measurements versus minimum temperature on day of sampling derived from 2208 pooled urine samples collected at Boonah, SEQ, Cairns, FNQ, and Yungaburra, FNQ (R^2^ = 0.83).

There was a strong inverse correlation between urinary cortisol concentration and minimum temperature on the day of sample collection (R^2^ = 0.83; p < 0.001), with the former increasing exponentially as temperature decreased (Fig 5). There was no significant association between urinary cortisol concentration and rainfall in the 24 hrs prior to sample collection (p = 0.45). Maximum daily temperature on the day previous to urine collection was also related to cortisol levels, but at a notably lower level (R^2^ = 0.35; p < 0.001), where cortisol concentration increased with decreasing maximum temperature. The relationship with both climatic variables was independent of region, total population size or species composition. Further, the relationships between cortisol measurements and environmental variables were consistent, whereby none of the environmental variables showed a significant interaction with region, population size or species composition. While total population size and species composition varied within and between regions over time, neither had a significant effect on cortisol concentration (p = 0.54 and p = 0.51 respectively).

Adjusted mean urinary cortisol concentration in Hendra virus RNA positive pooled urine samples was significantly higher than in Hendra virus RNA-negative samples (15.66 ± 1.15 ng/mL and 12.00 ± 1.11 ng/mL respectively; p = 0.006).

### Individual animal urine samples

**Fig 6:**
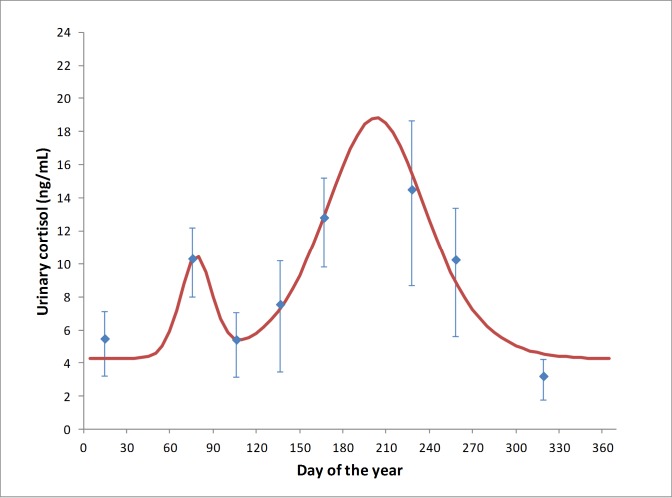
Mean monthly individual *P*. *alecto* urinary cortisol measurements across the 12-month study in SEQ demonstrating an estimated overall annual trend of urinary cortisol (R^2^ = 0.89).

Individual animal urine samples were collected from Boonah and Toowoomba roosts in SEQ (S1 Table). As all Hendra virus detections were in *P*. *alecto* (see below), the analyses were restricted to this species for parsimony. There was no significant difference between mean individual urinary cortisol concentration between collection events (p = 0.16), but there was an evident pattern (Fig 6) corresponding to the two statistically significant urinary cortisol concentration elevations reported for mean pooled population in autumn and winter in SEQ. No significant differences in mean urinary cortisol concentrations were demonstrated between male and female *P*. *alecto* or reproductive status (p = 0.54; 0.67 respectively), however females during birthing season (n = 17) had elevated urinary cortisol when compared with males at the same time of year (n = 7).

**Fig 7:**
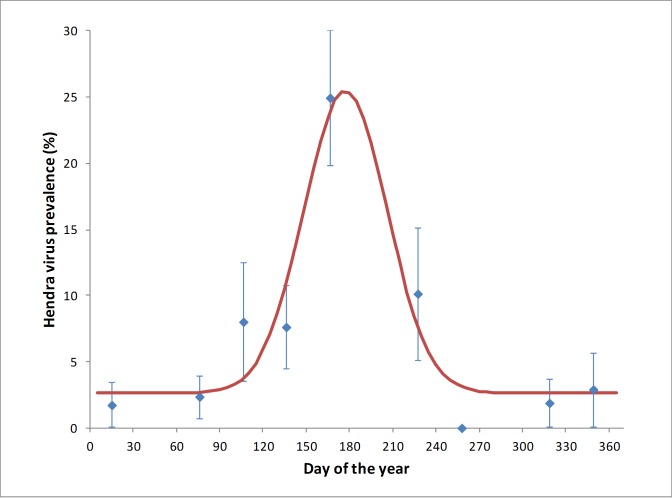
Mean individual *P*. *alecto* Hendra virus urinary excretion prevalence across the 12-month study in SEQ demonstrating an estimated overall annual trend of Hendra virus excretion prevalence (R^2^ = 0.90).

All Hendra virus RNA detections were in *P*. *alecto* samples (n = 34/375), with no detections in *P*. *poliocephalus* (n = 0/86) or *P*. *scapulatus* (n = 0/103) samples. The mean detection prevalence in *P*. *alecto* was 9.1%. Statistical analysis demonstrated a significant effect of month (p < 0.001) on RNA detection, with a significant elevation in excretion in June (24.9%) (Fig 7). Statistically non-significant elevations in excretion occurred in April, May and August, with Hendra virus RNA excretion prevalence of 8.0%, 7.6% and 10.1% respectively. In all other collection months, the excretion prevalence was below 3%. There was no significant difference in Hendra virus RNA excretion prevalence between sexes (p = 0.58), and no significant sex by month interaction (p = 0.60).

Urinary cortisol concentrations in individual *P*. *alecto* Hendra virus RNA-positive urine samples (n = 34) were slightly but non-significantly higher than those in *P*. *alecto* Hendra virus RNA-negative urine samples (n = 341) (p = 0.80).

## Discussion

Urine has previously been established as the primary route of excretion of Hendra virus [5], and as a suitable substrate for cortisol assay as an indicator of physiological stress in flying-foxes [24]. While plasma cortisol concentration theoretically provides a more immediate and sensitive measure of stress, the capture and handling stress associated with sample collection renders the approach impractical for a large-scale study. Our non-invasive under-roost sampling approach allowed the collection of both pooled and individual urine samples without the risk of distorting cortisol concentration, and facilitated the concurrent assessment of Hendra virus excretion status. It thus provided a valid assessment of temporal trends in urinary cortisol concentration at the population level (pooled samples) and individual bat level (individual samples), and the putative association between Hendra virus infection and physiological stress. Similarly, the use of molecular techniques to identify sex and species from individual urine samples allowed the investigation of the effects of life cycle on urinary cortisol concentration and Hendra virus urinary excretion without the potentially confounding effects of capture stress.

The collection of pooled urine samples presents few challenges logistically and analytically, and the potential bias consequent to an individual Hendra virus infected animal contributing to multiple pools (and erroneously inflating prevalence estimates) is mitigated by our systematic approach of collecting each pooled sample from a discrete section of each sheet. However, the under-roost sampling of individual animals can be problematic. Firstly, when roosting density is high, individual urination events are impossible to identify, and sampling opportunities have to be foregone. Secondly, the small volume of urine from the minimal number of drops collected to ensure that a sample is truly from a single individual limits the number of analyses that can be performed, and in some cases, provides only sufficient volume for molecular analysis. The latter might be overcome in the future with the emergence of molecular techniques for measuring physiological parameters, including those indicative of physiological stress, instead of current serological techniques that require a larger sample volume.

The pooled urine Hendra virus data used in our analyses were part of a larger study which showed, firstly, that *P*. *alecto* and *P*. *conspicillatus* were the primary Hendra virus reservoir species, secondly, that mean Hendra virus RNA urinary excretion prevalence was higher in SEQ than in FNQ, and thirdly, that virus excretion prevalence peaked in June, July, August (southern hemisphere winter) in lower latitude SEQ, while remaining more or less constant in FNQ [7]. The individual animal data from the current study showed the same winter pattern of virus excretion as the pooled population urine data in SEQ reported in that larger study [7].

While it would have been preferable to compare physiological responses in the same species in both SEQ and FNQ, the absence of *P*. *conspicillatus* in SEQ, their predominance in FNQ, and the frequent inaccessibility of *P*. *alecto* roosts in FNQ, precluded this. Thus, we used *P*. *conspicillatus* as a proxy for *P*. *alecto* in FNQ. The two species are phylogenetically paraphyletic [3], are morphologically similar and have synchronous life cycles [27], have no significant differences in cortisol profiles [24], and minimal difference in hematologic and biochemical reference ranges (L McMichael, unpublished data). Thus, we contend that their physiological changes in response to natural life cycle and seasonal stressors are comparable.

Levels of circulating glucocorticoids can vary with season, sex, age, social status and breeding stage, and valid interpretation of the data requires that these ‘natural’ variations are recognised [15]. Pooled urinary cortisol concentration was consistently elevated in March in the SEQ Boonah roost, and in February/March in both FNQ roosts, aligning with the February to April breeding season in *P*. *conspicillatus*, *P*. *alecto* and *P*. *poliocephalus* [27]. The literature suggests that this peak is associated with elevated cortisol levels in males, as a consequence of elevated testosterone levels and the physiological stress of territory defence and harem establishment [28, 29, 34]. However, the individual animal data showed that (in SEQ at least) the March elevations in cortisol were similar in both sexes, suggesting that the chaos of breeding season is equally stressful to females. Indeed, during the mating season females were regularly observed to exhibit signs of behavioural stress including agitated vocalisations that most likely constitute maternal contact calls in response to separation from their weaning pups. The greater elevation of cortisol in SEQ roosts (compared to FNQ roosts) may be plausibly explained by the synchronous breeding of *P*. *alecto* and *P*. *poliocephalus*, which in mixed roosts likely exacerbates stress through the combined effects of both intra- and interspecific interactions. However, we found no significant direct association between species and urinary cortisol concentration in the pooled urine models.

Pooled samples from the SEQ Boonah roost also showed elevations in urinary cortisol in June, July and August, corresponding with southern hemisphere winter, and with mid-late pregnancy in *P*. *alecto* and *P*. *poliocephalus*. No similar elevations were evident in either FNQ roost. While a peri-parturient cortisol spike is observed in well-studied domestic species [35], a pregnancy-associated elevation in cortisol mid-late gestation is unexpected in Pteropus species [20]. Further, given the phylogenetic proximity of *P*. *alecto* and *P*. *conspicillatus*, were the elevated cortisol associated with pregnancy, we would expect to see elevated levels in both SEQ and FNQ. Finally, the individual animal urine samples showed that the SEQ winter elevations in urinary cortisol were equally evident in both males and females, discounting an association with pregnancy. Thus, while the observed elevated urinary cortisol in autumn (common to both regions) is plausibly explained by the life-cycle event of mating, the winter elevation in SEQ is not associated with the subsequent event of pregnancy, and is more consistent with an environmental stressor impacting SEQ roosts but not FNQ roosts.

We identified a strong correlation between urinary cortisol concentration and minimum daily temperature in *P*. *alecto* populations in SEQ, and given the absence of evidence of an association with any other measured variable, we suggest that low ambient temperature is a biologically plausible stressor. While testing this proposition was beyond the scope of this study, minimum winter temperatures at the SEQ Boonah roost often fell below zero during the study period, with frosts occurring regularly; at the FNQ roosts, minimum temperatures rarely fell below 10°C. Further, veterinary examination of captured individual *P*. *alecto* at the SEQ Boonah roost in winter often revealed peripheral vasoconstriction and frost bite damage to ears and feet that was absent in co-roosting *P*. *poliocephalus*, a species historically with a more southerly range. Thus, we hypothesize that the recent extension of the range of *P*. *alecto* (historically a tropical species [36]) into sub-tropical and temperature regions, may promote physiological stress consequent to the physiological demand of winter thermoregulation. Lewanzik et al. [18] have previously reported an association between climatic factors and seasonal differences in cortisol levels in bats. Further, Hawley and Altizer [37] identify the physiological cost of winter thermoregulation as a cause of reduced immune system function, presenting a plausible explanation for the elevated Hendra virus infection prevalence in *P*. *alecto* in winter in SEQ [7]. To extrapolate further, McMichael et al., [38] showed a positive correlation between low plasma triglyceride levels, but no other nutritional biomarkers, and Hendra virus RNA excretion in *P*. *alecto*. While plasma triglyceride levels can be associated with a suite of factors, the thermogenic catabolism of triglycerides [39] to facilitate thermoregulation offers a plausible physiological mechanism. However, cortisol concentration measurement in our study was limited to species in which Hendra virus RNA was detected, consistent with our aim of exploring the relationship between Hendra virus infection and physiological stress. The demonstration of a contrasting winter cortisol concentration profile in *P*. *alecto* and *P*. *poliocephalus* in temperate roosts would further support our hypothesis that low ambient temperatures trigger physiological stress in *P*. *alecto*, leading to increased Hendra virus infection prevalence in winter in SEQ.

We found a small but statistically significant increase in urinary cortisol in pooled samples positive for Hendra virus RNA. In the individual animal urine samples, we found a small and non-significant increase in urinary cortisol measurements in Hendra virus RNA positive samples. This disparity is most plausibly explained by the greater temporal coverage and larger data set of the pooled sampling methodology. Edson et al. [25] similarly reported small but statistically significant increase in urinary cortisol in Hendra virus RNA positive pooled samples, but did not identify the nature or significance of the association.

## Conclusions

In this study, we sought to examine temporal trends in urinary cortisol concentration in disparate flying-fox populations and elucidate the relationship between Hendra virus infection and putative physiological stress. Our findings indicate that urinary cortisol excretion is modulated by both life cycle and ecological factors. We found no significant correlation between increased urinary cortisol due to normal life cycle events and Hendra virus excretion, but our findings do suggest a biologically plausible association between low winter temperatures and elevated cortisol levels in *P*. *alecto* in the lower latitude SEQ roosts. Based on our findings and previous studies, we hypothesize an association between low winter temperatures and increased Hendra virus infection and excretion, putatively mediated by the physiological cost of thermoregulation. Further work is required to elaborate and test this hypothesis.

While this study has focused on Australian pteropids and Hendra virus, our findings and our approach have direct relevance to studies of the disease ecology of Nipah virus and other emerging Henipaviruses in bats. More broadly, they inform investigation of emerging disease infection dynamics at any wildlife/livestock/human interface.

## Supporting information

S1 Text Species and sex differentiation primers. (DOCX)

S1 Table Pooled and individual animal urine samples. (DOCX)
