# Supplementary material for: Physiological stress and Hendra virus in flying-foxes (Pteropus spp.), Australia
Source: PLoS One. 2017 Aug 2;12(8):e0182171. doi: 10.1371/journal.pone.0182171 (PMC5540484; doi:10.1371/journal.pone.0182171)
Supplement: S1 Text — (DOCX) [file pone.0182171.s001.docx]

Species differentiation primer and probe sequences are as follows: *P. alecto/P. conspicillatus* forward primer BSFFCytBF (CAT GCT AAC GGA GCA TCC AT), reverse primer BSFFCytBR (ACA CCT ACG TTT CAG GTT TCT TTG) and probe BSFFCytB-FAMMGB (FAM-ACA TGT AGG CCG AGG C-MGB); *P. poliocephalus* forward primer GHFFCytBF (CAT CTG CCT ATT CCT GCA TGT G), reverse primer GHFFCytBR (GCA AAT AGA AGG ATG ACA CCT ACG T) and probe GHFFCytB-VICMGB (VIC-ATC TTA CAT CTA TAA AGA GAC CTG A-MGB); *P. scapulatus* forward primer LRFFCytB (GGA GCG TCC ATA TTC TTT ATC TG), reverse primer LRFFCytBR (TTA CGG CAA ATA GGA GGA TAA CG) and probe LRFFCytB-NEDMGB (NED-ACA CGT AGG CCG AGG C-MGB). Sex differentiation primer and probe sequences are as follows: forward primer SRY-F (GCA AGG CGG CTC TAG AAA ATC), reverse primer SRY-R (TCC CAG TCG CTT GCT GAT C) and probe SRY-NEDMGB (NED-CAA AAT GCA AAA CTC GG-MGB).
