# Supplementary material for: Physiological stress and Hendra virus in flying-foxes (Pteropus spp.), Australia
Source: PLoS One. 2017 Aug 2;12(8):e0182171. doi: 10.1371/journal.pone.0182171 (PMC5540484; doi:10.1371/journal.pone.0182171)
Supplement: S1 Table — (DOCX) [file pone.0182171.s002.docx]

| **Sample Type** | **Region** | **Location** | **Date** | **Pteropus Species** | **Number** |
| --- | --- | --- | --- | --- | --- |
| Pooled | SEQ | Boonah | 12/01/2012 | *P. alecto,*  *P. poliocephalus* | 29 |
| Pooled | SEQ | Boonah | 02/02/2012 | *P. alecto,*  *P. poliocephalus* | 30 |
| Pooled | SEQ | Boonah | 09/03/2012 | *P. alecto,*  *P. poliocephalus* | 30 |
| Pooled | SEQ | Boonah | 13/04/2012 | *P. alecto,*  *P. poliocephalus* | 30 |
| Pooled | SEQ | Boonah | 04/05/2012 | *P. alecto,*  *P. poliocephalus* | 30 |
| Pooled | SEQ | Boonah | 17/05/2012 | *P. alecto,*  *P. poliocephalus* | 30 |
| Pooled | SEQ | Boonah | 15/06/2012 | *P. alecto,*  *P. poliocephalus* | 30 |
| Pooled | SEQ | Boonah | 04/07/2012 | *P. alecto,*  *P. poliocephalus* | 30 |
| Pooled | SEQ | Boonah | 08/08/2012 | *P. alecto,*  *P. poliocephalus* | 30 |
| Pooled | SEQ | Boonah | 11/09/2012 | *P. alecto,*  *P. poliocephalus* | 30 |
| Pooled | SEQ | Boonah | 10/10/2012 | *P. alecto,*  *P. poliocephalus* | 30 |
| Pooled | SEQ | Boonah | 13/11/2012 | *P. alecto,*  *P. poliocephalus* | 30 |
| Pooled | SEQ | Boonah | 14/12/2012 | *P. alecto,*  *P. poliocephalus* | 60 |
| Pooled | SEQ | Boonah | 03/01/2013 | *P. alecto,*  *P. poliocephalus* | 30 |
| Pooled | SEQ | Boonah | 07/02/2013 | *P. alecto,*  *P. poliocephalus,*  *P. scapulatus* | 30 |
| Pooled | SEQ | Boonah | 12/03/2013 | *P. alecto,*  *P. poliocephalus,*  *P. scapulatus* | 30 |
| Pooled | SEQ | Boonah | 02/05/2013 | *P. alecto,*  *P. poliocephalus* | 30 |
| Pooled | SEQ | Boonah | 05/06/2013 | *P. alecto,*  *P. poliocephalus* | 30 |
| Pooled | SEQ | Boonah | 10/07/2013 | *P. alecto,*  *P. poliocephalus* | 30 |
| Pooled | SEQ | Boonah | 07/08/2013 | *P. alecto,*  *P. poliocephalus* | 30 |
| Pooled | SEQ | Boonah | 04/09/2013 | *P. alecto,*  *P. poliocephalus* | 30 |
| Pooled | SEQ | Boonah | 26/09/2013 | *P. alecto,*  *P. poliocephalus* | 31 |
| Pooled | SEQ | Boonah | 22/10/2013 | *P. alecto,*  *P. poliocephalus,*  *P. scapulatus* | 60 |
| Pooled | SEQ | Boonah | 03/12/2013 | *P. alecto,*  *P. poliocephalus,*  *P. scapulatus* | 59 |
| Pooled | SEQ | Boonah | 15/01/2014 | *P. alecto,*  *P. poliocephalus* | 25 |
| Pooled | SEQ | Boonah | 17/01/2014 | *P. alecto,*  *P. poliocephalus* | 60 |
| Pooled | SEQ | Boonah | 11/02/2014 | *P. alecto,*  *P. poliocephalus,*  *P. scapulatus* | 60 |
| Pooled | SEQ | Boonah | 11/03/2014 | *P. alecto,*  *P. poliocephalus* | 56 |
| Pooled | SEQ | Boonah | 30/04/2014 | *P. alecto,*  *P. poliocephalus* | 49 |
| Pooled | SEQ | Boonah | 30/05/2014 | *P. alecto,*  *P. poliocephalus* | 60 |
| Pooled | FNQ | Cairns | 10/05/2012 | *P. conspicillatus* | 30 |
| Pooled | FNQ | Cairns | 11/07/2012 | *P. conspicillatus* | 30 |
| Pooled | FNQ | Cairns | 01/08/2012 | *P. conspicillatus* | 60 |
| Pooled | FNQ | Cairns | 05/09/2012 | *P. conspicillatus* | 30 |
| Pooled | FNQ | Cairns | 04/10/2012 | *P. conspicillatus* | 30 |
| Pooled | FNQ | Cairns | 08/11/2012 | *P. conspicillatus* | 30 |
| Pooled | FNQ | Cairns | 06/12/2012 | *P. conspicillatus,*  *P. scapulatus* | 30 |
| Pooled | FNQ | Cairns | 10/01/2013 | *P. conspicillatus,*  *P. scapulatus* | 30 |
| Pooled | FNQ | Cairns | 14/02/2013 | *P. conspicillatus* | 30 |
| Pooled | FNQ | Cairns | 19/03/2013 | *P. conspicillatus* | 30 |
| Pooled | FNQ | Cairns | 16/04/2013 | *P. conspicillatus* | 30 |
| Pooled | FNQ | Cairns | 07/05/2013 | *P. conspicillatus* | 30 |
| Pooled | FNQ | Cairns | 04/06/2013 | *P. conspicillatus* | 24 |
| Pooled | FNQ | Cairns | 13/08/2013 | *P. conspicillatus* | 30 |
| Pooled | FNQ | Cairns | 12/09/2013 | *P. conspicillatus* | 40 |
| Pooled | FNQ | Cairns | 12/10/2013 | *P. conspicillatus,*  *P. scapulatus* | 60 |
| Pooled | FNQ | Cairns | 05/12/2013 | *P. conspicillatus* | 60 |
| Pooled | FNQ | Cairns | 27/02/2014 | *P. conspicillatus* | 60 |
| Pooled | FNQ | Cairns | 18/03/2014 | *P. conspicillatus,*  *P. alecto* | 60 |
| Pooled | FNQ | Cairns | 29/04/2014 | *P. conspicillatus* | 58 |
| Pooled | FNQ | Cairns | 25/06/2014 | *P. conspicillatus* | 44 |
| Pooled | FNQ | Yungaburra | 03/04/2012 | *P. conspicillatus* | 30 |
| Pooled | FNQ | Yungaburra | 09/05/2012 | *P. conspicillatus* | 30 |
| Pooled | FNQ | Yungaburra | 07/11/2012 | *P. conspicillatus* | 30 |
| Pooled | FNQ | Yungaburra | 05/12/2012 | *P. conspicillatus* | 30 |
| Pooled | FNQ | Yungaburra | 09/01/2013 | *P. conspicillatus,*  *P. scapulatus* | 30 |
| Pooled | FNQ | Yungaburra | 13/02/2013 | *P. conspicillatus,*  *P. scapulatus* | 60 |
| Pooled | FNQ | Yungaburra | 20/03/2013 | *P. conspicillatus* | 30 |
| Pooled | FNQ | Yungaburra | 17/04/2013 | *P. conspicillatus* | 30 |
| Individual | SEQ | Toowoomba | 29/03/2013 | *P. alecto* | 88 |
| Individual | SEQ | Toowoomba | 29/03/2013 | *P. poliocephalus* | 15 |
| Individual | SEQ | Boonah | 26/06/2013 | *P. alecto* | 73 |
| Individual | SEQ | Boonah | 26/06/2013 | *P. poliocephalus* | 5 |
| Individual | SEQ | Boonah | 21/08/2013 | *P. alecto* | 45 |
| Individual | SEQ | Boonah | 21/08/2013 | *P. poliocephalus* | 5 |
| Individual | SEQ | Boonah | 01/09/2013 | *P. alecto* | 24 |
| Individual | SEQ | Boonah | 01/09/2013 | *P. poliocephalus* | 9 |
| Individual | SEQ | Boonah | 08/11/2013 | *P. alecto* | 27 |
| Individual | SEQ | Boonah | 08/11/2013 | *P. poliocephalus* | 4 |
| Individual | SEQ | Boonah | 08/11/2013 | *P. scapulatus* | 42 |
| Individual | SEQ | Boonah | 18/12/2013 | *P. alecto* | 3 |
| Individual | SEQ | Boonah | 18/12/2013 | *P. scapulatus* | 40 |
| Individual | SEQ | Boonah | 04/01/2014 | *P. alecto* | 36 |
| Individual | SEQ | Boonah | 04/01/2014 | *P. poliocephalus* | 2 |
| Individual | SEQ | Boonah | 04/01/2014 | *P. scapulatus* | 21 |
| Individual | SEQ | Boonah | 30/04/2014 | *P. alecto* | 42 |
| Individual | SEQ | Boonah | 30/04/2014 | *P. poliocephalus* | 23 |
| Individual | SEQ | Boonah | 30/05/2014 | *P. alecto* | 37 |
| Individual | SEQ | Boonah | 30/05/2014 | *P. poliocephalus* | 23 |
